# Chondroblastoma of the distal femur resected through a small fenestra via computed tomography navigation and endoscopy: a case report

**DOI:** 10.1186/1752-1947-7-164

**Published:** 2013-06-27

**Authors:** Tsuyoshi Miyazaki, Kenzo Uchida, Takafumi Yayama, Hideaki Nakajima, Kazuya Honjoh, Hiroshi Itoh, Yoshinao Oda, Hisatoshi Baba

**Affiliations:** 1 Department of Orthopaedics and Rehabilitation Medicine, Faculty of Medical Sciences, University of Fukui, Matsuoka Shimoaizuki 23-3, Eiheiji, Fukui 910-1193, Japan; 2 Department of Molecular Pathology, Yamaguchi University Graduate School of Medicine, Yamaguchi, Japan; 3 Department of Anatomic Pathology, Graduate School of Medical Sciences, Kyushu University, Fukuoka, Japan

## Abstract

**Introduction:**

Chondroblastoma is a benign bone tumor with a relatively high incidence in older children and adolescents during the period of active epiphyseal growth. It is generally regarded as a benign neoplasm, but sometimes it grows aggressively or recurs. To prevent recurrence, complete curettage is important; however, such an approach can be extremely difficult to perform precisely when the chondroblastoma arises deep in the epiphysis. In our patient’s case, we used a computed tomography-based navigation system with registration technique involving skin marker fiduciaries and endoscopic curettage of the lesion.

**Case presentation:**

A 16-year-old Japanese girl presented to our facility with left knee joint pain, which started nine months before her initial examination. Computed tomography and magnetic resonance imaging studies of the left knee showed a radiolucent lesion with marginal sclerosis and lobular homogeneous hypo-intensity and hyper-intensity signals in the distal epiphysis of the left femoral epiphysis, carried through to the growth plate. To prevent recurrence of chondroblastoma and growth disturbance, we used a computed tomography-based navigation system with registration technique involving skin marker fiduciaries and endoscopic curettage of the lesion. Wide excision with total removal of the chondroblastoma in the distal femur often requires large exposure with associated drawbacks, where a wide excision near the growth plate can potentially lead to growth disturbance. Therefore, in an accessible location in the distal femur, endoscopic excision of chondroblastoma under navigation system guidance can be performed with minimal operative damage.

**Conclusions:**

In the setting of a benign intra-osseous lesion infiltrating the growth plate, arthroscopic retrieval or excision under a computed tomography-based navigation system should be considered before proceeding with open surgery.

## Introduction

Chondroblastoma is a benign bone tumor with a relatively high incidence in older children and adolescents during the period of active epiphyseal growth. Although it is generally regarded as a benign neoplasm, it sometimes grows aggressively or recurs; rarely, it metastasizes to the lungs. To prevent recurrence, complete curettage is important; however, such an approach becomes extremely difficult to perform precisely when the chondroblastoma arises deep in the epiphysis. Additionally, the damage to the growth plate can induce growth disturbance and deformities. Recently, some groups reported the usefulness of a computed tomography (CT)-based navigation system and endoscopy for curettage and resection of osseous lesions [1-4]. The present report describes a minimally invasive approach using not only a CT-based navigation system but also endoscopy for chondroblastoma curettage in the distal femoral epiphysis.

## Case presentation

**Figure 1:**
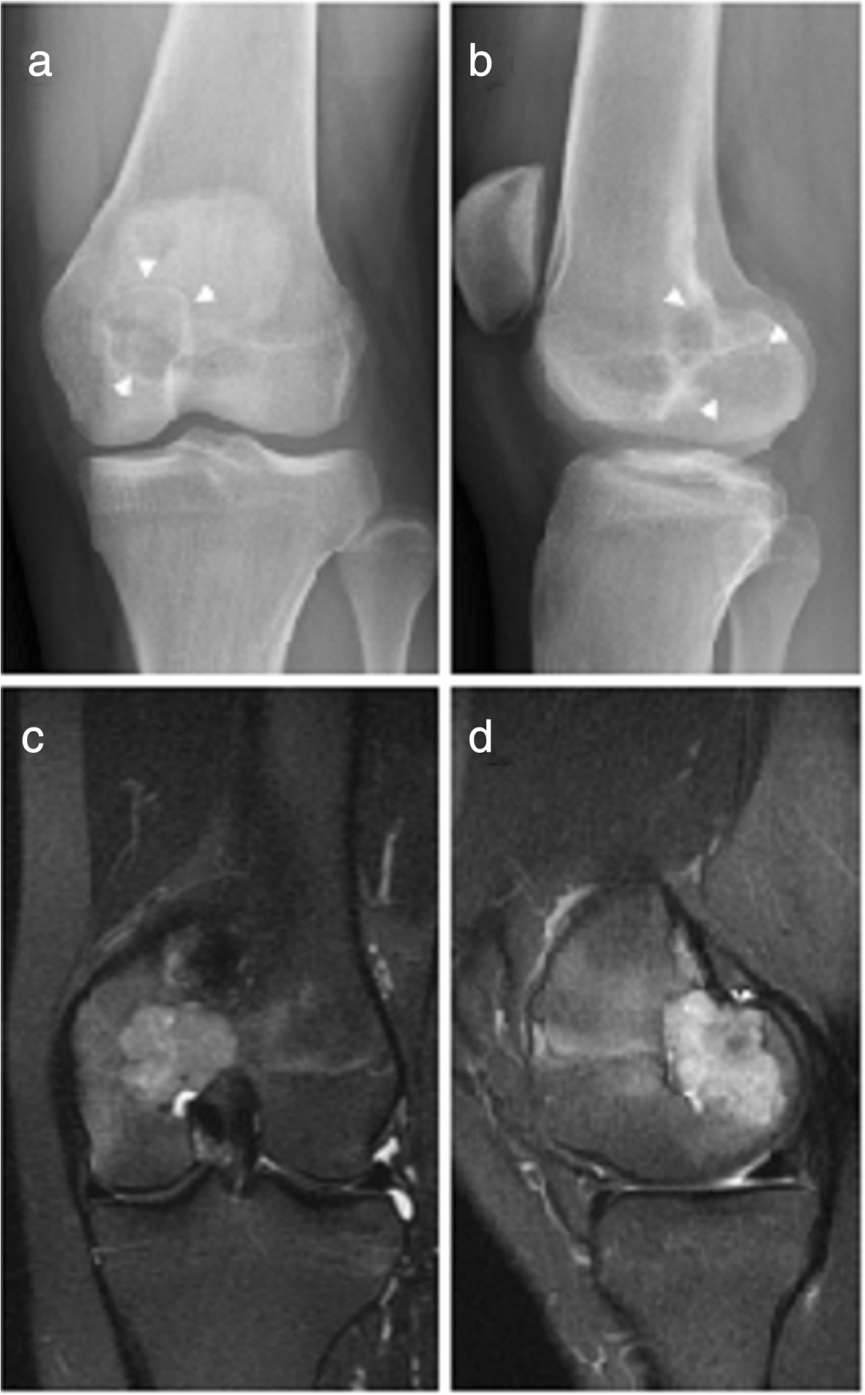
Lesion in the distal epiphysis of the left femur showed marginal sclerosis on radiographs (a,b:arrowheads) and bone edema in magnetic resonance T1-weighted Gd-enhanced images (c,d).

A 16-year-old Japanese girl who was a high school student noticed left knee joint pain with joint motion and a decrease in weight-bearing ability, which started nine months before her initial presentation to our facility. On physical examination, joint effusion, spontaneous pain and tenderness in the medial side of the left knee were noted. The full range of motion of the left knee was preserved; there was no color change or redness of the overlying skin. Laboratory test results showed no abnormalities. Radiographs of the left knee showed a radiolucent lesion with marginal sclerosis in the distal epiphysis of the left femur (Figure 1a,b). On magnetic resonance imaging (MRI) (1.5T Signa; General Electric Medical Systems, Milwaukee, WI, USA), the T1-weighted (repetition time (TR) 417ms, echo time (TE) 8ms) spin-echo image enhanced by gadolinium-diethylenetriaminepenta-acetic acid (Gd-EDTA) showed hyper-intensity and iso-intensity signals with bone edema around the lesion (Figure 1c,d). Computed tomography of the left knee showed no link between the tumor cavity and joint space.

A histopathological examination of a small incisional biopsy conducted before operation was suggestive of chondroblastoma. The lesion was found to be deep to the medial condyle; it spread beyond the growth plate and contacted the origin of the posterior cruciate ligament of the knee. We planned to use a navigation system for precise curettage of the lesion, in addition to endoscopy to view the lesion directly through the small fenestra of the bone.

**Figure 2:**
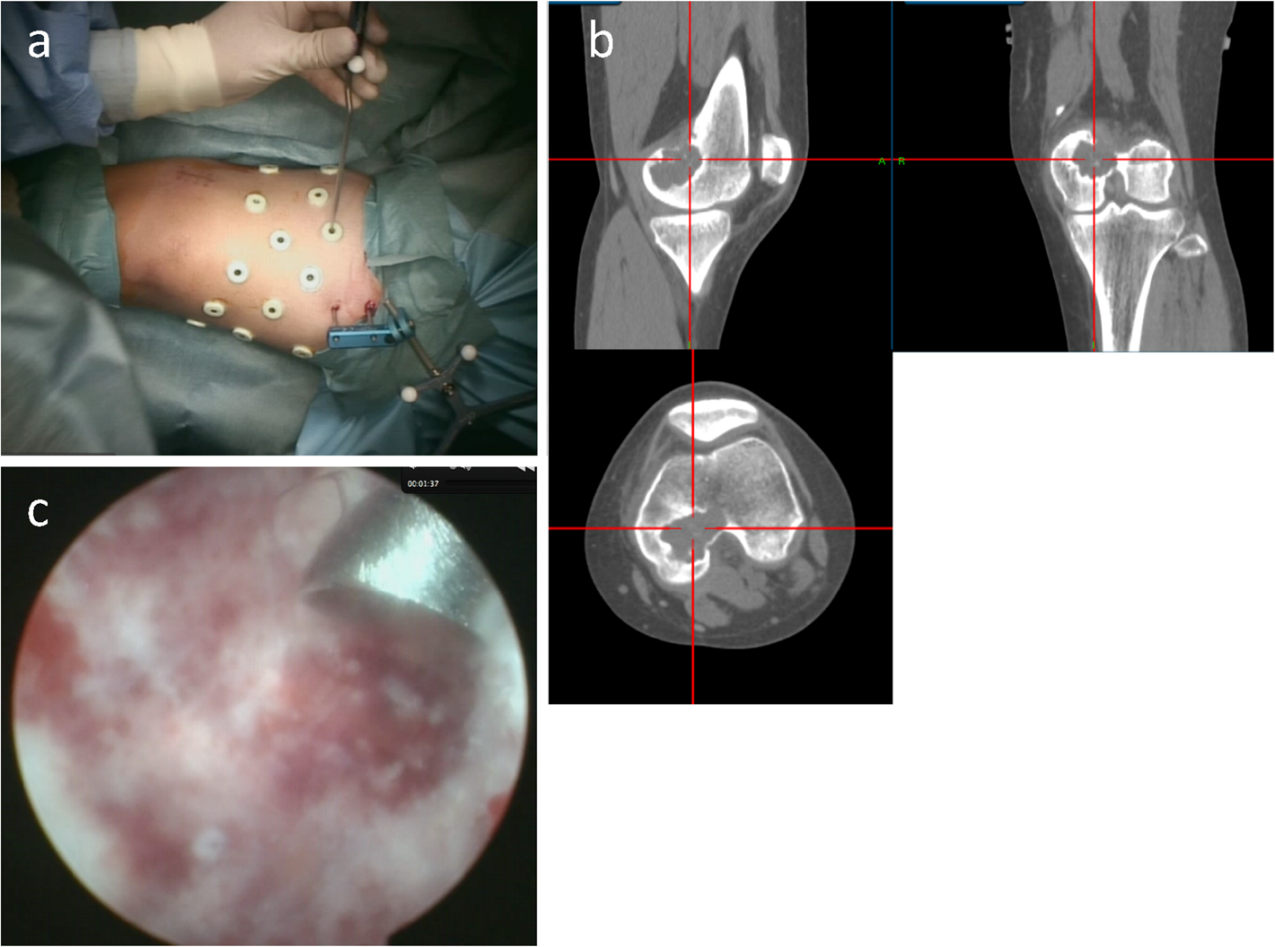
Registration of navigation system using multiple skin-point markers (a) to confirm curetted area (b) and endoscopic view of the curetted lesion (c).

The Stealth Station® Tria® Navigation System (Medtronic Navigation, Inc, Louisville, CO, USA) was used for computer-navigation surgical treatment. This navigation system consists of a computer workstation, a reference frame with passive markers, a standard probe, and an electro-optical camera connected to the computer workstation that serves as a position sensor. Since no specific application has been developed to support the resection of tumors, we used the ‘Spine’ module developed for pedicle screw application. The system uses CT data to set the region of interest. For routine CT-based navigation, paired point-based registration uses fiduciary markers that are fixed invasively to the surface of the involved bone. In the current study, multiple skin-point markers were used to avoid such an invasive marking procedure (Figure 2a).

**Figure 3:**
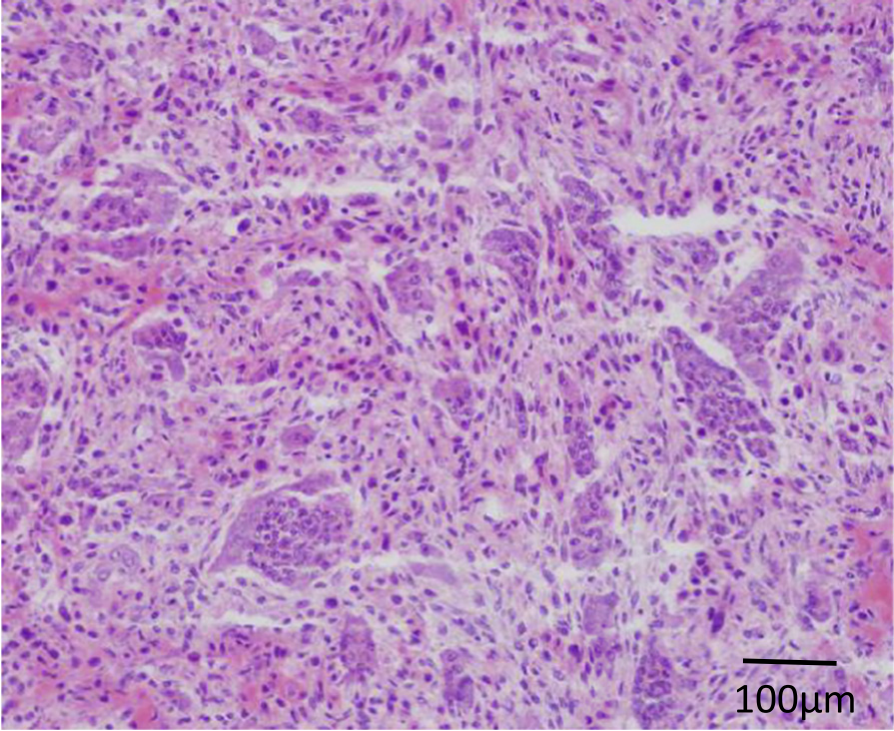
Microscopic findings of the surgical specimen (hematoxylin and eosin staining, ×20) showing a cellular lesion with chondroid stromal production and calcification assuming a fine linear pattern.

Knee arthroscopy was performed first to examine the posterior cruciate ligament origin; however, no apparent communication was found between the medial compartment of the knee joint and the osseous lesion. Under navigation system guidance, a small skin incision (2cm in length) was made over the medial femoral condyle. A small fenestration hole (approximately 1.5cm in diameter) was made on the femoral cortex. Using the navigation system, curettage of the lesion was performed, with confirmation of the curettage area (Figure 2b). Additional curettage and abrasion of the lesion were undertaken using the endoscope (Figure 2c). Histopathology results showed proliferation of oval short-spindle cells and osteoclast-like multinucleated giant cells, accompanied by blue-colored or pink-colored chondroid matrix. Focal osteoid formations were also observed (Figure 3).

**Figure 4:**
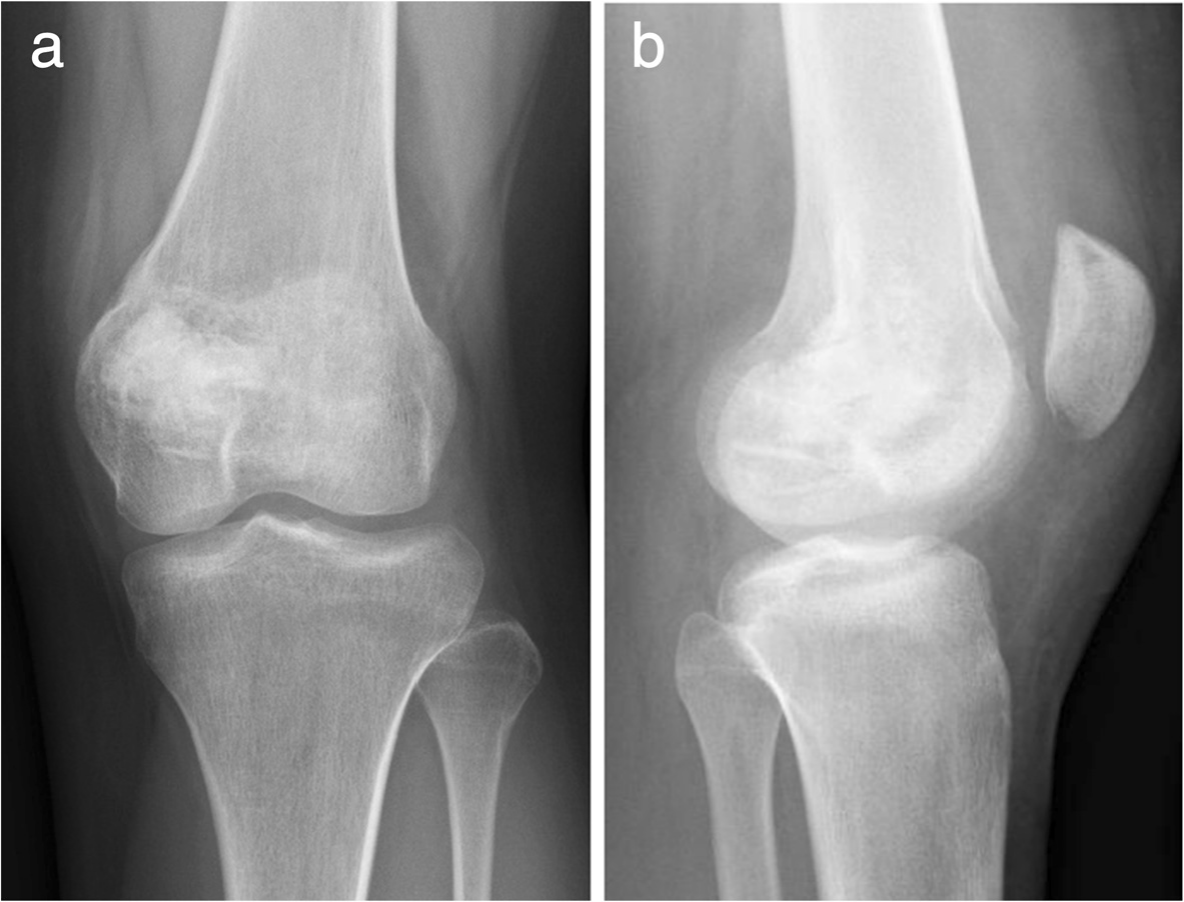
Radiographs at one-year follow-up, showing bone remodeling and consolidation (a,b).

After the CT-based navigation and endoscopy for curettage of the lesion, the defect was completely filled with autograft bone and β-tricalcium phosphate granules (OSferion® Olympus, Tokyo, Japan). Our patient’s post-operative course was uneventful, and follow-up radiography showed excellent filling of the autograft and artificial bone in the medial femoral condyle. Our patient is doing well at one year after surgery (Figure 4).

## Discussion

There are no data on the growth disturbance rates and growth deformities after treatment of bone tumor around the growth plate. One study found 7 percent (6 of 81) of patients with minor shortening, all in patients aged 14 years or younger with open growth plates, though they did not specify the involved site [5]. Another study noted that few patients with chondroblastoma had much growth remaining, but that four patients who had treatment before skeletal maturity had experienced growth plate disturbances [6]. In our patient’s case, the tumor carried through the growth plate and we were able to curette the lesion through a small fenestra in the upper part of the medial condyle under CT-based navigation system guidance to avoid bone loss around the lesion and growth plate injury, and additional curettage and abrasion were undertaken via endoscopy without extensive bone loss.

To avoid recurrence, good visualization of the chondroblastoma lesion is essential for complete curettage [7-9]. High-intensity illumination of the magnified arthroscopic image provides excellent visibility of the tumor cavity. In our patient’s case, we were able to view the entire internal area of the lesion through a small fenestra with ‘wet’ arthroscopic viewing (using continuous irrigation). Consequently, there was minimal interference from bleeding from the walls of the lesion and curettage and abrasion of the cyst wall were conducted under direct view.

In the present case, the combined use of a CT-based navigation system and arthroscopy offered certain advantages. First, multiple adjacent levels automatically registered simultaneously and we were able to confirm the adequacy of the curetted area by navigation and arthroscopy. Second, there was minimal extensive bone loss in the lesion. Third, the use of a CT-based navigation system allowed us to curette and remove all the cystic cavities precisely inside the main lesion. Although computer-navigation surgery can accurately localize the lesion, there still may be errors. Inaccuracy of the points has been reported when CT was used, with differences up to 2 to 4mm at the time of surgery [10]. To avoid such a problem, we increased the skin markers by 50 percent and covered the entire knee with a plastic film until pre-operative registration in order to reduce the registration loss. In our patient’s case, the difference in the registration was approximately 1.8mm.

## Conclusions

In the setting of a benign intra-osseous lesion infiltrating the growth plate, arthroscopic retrieval or excision under CT-based navigation system guidance should be considered before proceeding with open surgery.

## Consent

Written informed consent was obtained from the patient’s parent/legal guardian for publication of this manuscript and any accompanying images. A copy of the written consent is available for review by the Editor-in-Chief of this journal.

## Competing interests

The authors declare that they have no competing interests.

## Authors’ contributions

TM, KU, TY, HN, KH, HI and YO made substantial contributions to the acquisition and interpretation of data. HB gave final approval of the version to be published. All authors read and approved the final manuscript.

## Authors’ information

All authors are specialized in the diagnosis and treatment of bone tumors. Research projects of our team include clinical and basic research for bone tumor treatment.
